# Mapping Haplotype-haplotype Interactions with Adaptive LASSO

**DOI:** 10.1186/1471-2156-11-79

**Published:** 2010-08-27

**Authors:** Ming Li, Roberto Romero, Wenjiang J Fu, Yuehua Cui

**Affiliations:** 1 Department of Epidemiology, Michigan State University, East Lansing, Michigan 48824, USA; 2 Department of Statistics and Probability, Michigan State University, East Lansing, Michigan 48824, USA; 3 The Perinatology Research Branch, NICHD, NIH, DHHS, Bethesda, MD, and Detroit, MI 48201, USA

## Abstract

**Background:**

The genetic etiology of complex diseases in human has been commonly viewed as a complex process involving both genetic and environmental factors functioning in a complicated manner. Quite often the interactions among genetic variants play major roles in determining the susceptibility of an individual to a particular disease. Statistical methods for modeling interactions underlying complex diseases between single genetic variants (e.g. single nucleotide polymorphisms or SNPs) have been extensively studied. Recently, haplotype-based analysis has gained its popularity among genetic association studies. When multiple sequence or haplotype interactions are involved in determining an individual's susceptibility to a disease, it presents daunting challenges in statistical modeling and testing of the interaction effects, largely due to the complicated higher order epistatic complexity.

**Results:**

In this article, we propose a new strategy in modeling haplotype-haplotype interactions under the penalized logistic regression framework with adaptive *L*_1_-penalty. We consider interactions of sequence variants between haplotype blocks. The adaptive *L*_1_-penalty allows simultaneous effect estimation and variable selection in a single model. We propose a new parameter estimation method which estimates and selects parameters by the modified Gauss-Seidel method nested within the EM algorithm. Simulation studies show that it has low false positive rate and reasonable power in detecting haplotype interactions. The method is applied to test haplotype interactions involved in mother and offspring genome in a small for gestational age (SGA) neonates data set, and significant interactions between different genomes are detected.

**Conclusions:**

As demonstrated by the simulation studies and real data analysis, the approach developed provides an efficient tool for the modeling and testing of haplotype interactions. The implementation of the method in R codes can be freely downloaded from http://www.stt.msu.edu/~cui/software.html.

## Background

It has been commonly recognized that most human diseases are complex involving joint effort of multiple genes, complicated gene-gene as well as gene-environment interactions [1]. The identification of disease risk factors for monogenic diseases has been quite successful in the past. Due to the small effect of many single genetic variants on the risk of a disease, the identification of disease variants for complex multigenic diseases has not been very successful [2]. There are multiple reasons for this. First, most complex diseases involve multiple genetic variants each conferring a small or moderate effect on a disease risk. Second, the complexity relies on the complicated interactions among disease variants, on a single-single variants or multiple-multiple variants basis. Third, but not the last, gene-environment interaction also plays pivotal roles in determining the underlying complexity of disease etiology. Studies on testing gene-gene interactions have been commonly pursued in the past, but little has been achieved, despite its importance in determining a disease risk (see [3] for a comprehensive review).

Mapping genetic interactions has been traditionally pursued in model organisms to identify functional relationships among genes [4-6]. With the seminal work in quantitative trait loci (QTL) mapping by Lander and Botstein [7], extensive work has been focused on experimental crosses to study the genetic architecture of complex traits. Along the line, methods for mapping QTL interactions have also been developed [8,9]. The recent development of human HapMap and radical breakthrough in genotyping technology have enabled us to generate high throughput single nucleotide polymorphisms (SNPs) data which are dense enough to cover the whole genome [10]. This advancement allows us to characterize variants at a sequence level that encode a complex disease phenotype, and opens a prospective future for disease variants identification [11,12].

Genetic interaction, or termed epistasis, occurs when the effect of one genetic variant is suppressed or enhanced by the existence of other genetic variants [13]. In align with this definition, Mani *et al*. [14] recently defined two distinct genetic interactions, namely the *synergistic interaction *in which extreme phenotype is expected whenever double mutations are present, and the *alleviating interaction *where one mutation in one gene masks the effect of another mutation by impairing the function of relative pathways. As an important component of the genetic architecture of many biological traits, the role of epistasis in shaping an organism's development has been unanimously recognized [15,16]. An increasing number of empirical studies have also revealed the role of epistasis in the pathogenesis of most common human diseases, such as cancer or cardiovascular disease [17,18].

The high-dimensional SNP data present unprecedented opportunities as well as daunting challenges in statistical modeling and testing in identifying genetic interactions. However, for most complex diseases, it remains largely unknown which combination of genetic variants is causal to the disease. Given that most traits or diseases are multifactorial and genetically complex, it is very unlikely that the function of a single variant can induce an overt disease signal without modeling the gene networks or pathways. Lin and Wu [19] proposed a sequence interaction model in a linear regression framework for a quantitative phenotype. Zhang *et al*. [20] proposed an entropy-based method for searching haplotype-haplotype interactions using unphased genotype data with applications in type I diabetes. Musani *et al*. [21] and Cordell [3] recently gave a comprehensive review of statistical methods developed for detecting gene-gene interactions. While most methods are nonparametric in nature such as the popular multifactor dimensionality reduction (MDR) method [22], they do not provide effect estimates for gene-gene interactions. Thus methods focusing on data reduction ignore the biological interpretation of the interaction. For instance, if two SNPs are identified to have interaction, how do they interact in genetics? What are the modes of gene action?

In Cui *et al*. [12], a novel approach was proposed to group haplotypes to detect risk haplotypes associated with a disease. In an extension to this work, we proposed a new statistical method to model haplotype-haplotype interactions responsible for a binary disease phenotype. We assume a population-based case-control design where a disease phenotype is assumed dichotomous. Due to high-order interactions, we propose a penalized logistic regression framework with adaptive *L*_1_-penalty, commonly termed as the adaptive LASSO [23]. The adaptive *L*_1_-penalty allows effect estimation and variable selection simultaneously in a single model. Moreover, it preserves the oracle property of variable selection [23]. Due to the binary nature of the response, we proposed a modified Gauss-Seidel method nested within the EM algorithm to estimate parameters. The model is applied to a real data set in which significant haplotype interactions are detected between mother and offspring genomes that might be responsible for disease risks in pregnancy.

## Methods

We first explain our method for a model involving interactions of haplotypes in 2 different haplotype blocks containing 2 SNPs in each. More complex models could be easily extended. Assume we have a study sample of *n *unrelated subjects with *n*_1 _cases and *n*_2 _controls. A number of SNPs are genotyped either in a genome-wide scale or in a candidate gene-based scale. Following the notation given in Liu *et al*. [11] and Cui *et al*. [12], we construct composite diplotypes by defining a distinct haplotype termed as "risk" haplotype for each haplotype block. Assuming two SNPs in each block, there could be nine possible genotypes, numerically denoted as 11/11, 11/12, 11/22, 12/11, 12/12, 12/22, 22/11, 22/12, 22/22. Without loss of generality, we assume [11] to be the "risk" haplotype. We denote the risk haplotype as *H *and all other non-risk haplotype as $\bar{H}$. In doing so, we can map the observed genotypes to three possible composite diplotypes, i.e., *HH*, $H\bar{H}$ and $\bar{H}\bar{H}$. Except for the double heterozygote 12/12 which is phase ambiguous and could be from two possible composite diplotypes, all other genotypes can be mapped to unique composite diplotypes. A detailed list of the configuration is given in Table 1.

**Table 1:** The configuration of two SNP combinations

Observed Genotype	Diplotype			Composite Diplotype
		
	Configuration	Frequency	**Relative Freq**.	
11/11	[11][11]	$p_{11}^{2}$	1	*HH*
11/12	[11][12]	2 *p*_11_*p*_12_	1	$H\bar{H}$
11/22	[12][12]	$p_{12}^{2}$	1	$\bar{H}\bar{H}$
12/11	[11][21]	2 *p*_11_*p*_21_	1	$\bar{H}\bar{H}$
12/12	$\left\{ \begin{array}{l} \left[11][22\right]\\ \left[12][21\right] \end{array}\right.$	$\left\{ \begin{array}{l} p_{11}p_{22}\\ p_{12}p_{21} \end{array}\right.$	$\left\{ \begin{array}{l} \phi \\ 1-\phi \end{array}\right.$	$\left\{ \begin{array}{l} H\bar{H}\\ \bar{H}\bar{H} \end{array}\right.$
12/22	[12][22]	2 *p*_12_*p*_22_	1	$\bar{H}\bar{H}$
22/11	[21][21]	$p_{21}^{2}$	1	$\bar{H}\bar{H}$
22/12	[21][22]	2 *p*_21_*p*_22_	1	$\bar{H}\bar{H}$
22/22	[22][22]	$p_{22}^{2}$	1	$\bar{H}\bar{H}$

Where $\phi =\frac{p_{11}p_{22}}{p_{11}p_{22}+p_{12}p_{21}}$

### The epistasis model

We consider two haplotype blocks *s *and *t*, each with two SNPs. There are total 81 possible genotype combinations. In each block, only the double heterozygote has ambiguous linkage phase, thus 64 genotypes could be mapped to unique composite diplotypes. Let (*H*_1_, ${\bar{H}}_{1}$) and (*H*_2_, ${\bar{H}}_{2}$) be the risk and non-risk haplotypes at blocks *s *and *t*, respectively. Expressed in terms of composite diplotypes, the four haplotypes can form nine distinct composite diplotypes expressed as *H*_1_*H*_1_*H*_2_*H*_2 _, $H_{1}{\bar{H}}_{1}H_{2}H_{2}$, ${\bar{H}}_{1}{\bar{H}}_{1}H_{2}H_{2}$, $H_{1}H_{1}H_{2}{\bar{H}}_{2}$, $H_{1}{\bar{H}}_{1}H_{2}{\bar{H}}_{2}$, ${\bar{H}}_{1}{\bar{H}}_{1}H_{2}{\bar{H}}_{2}$, $H_{1}H_{1}{\bar{H}}_{2}{\bar{H}}_{2}$, $H_{1}{\bar{H}}_{1}{\bar{H}}_{2}{\bar{H}}_{2}$ and ${\bar{H}}_{1}{\bar{H}}_{1}{\bar{H}}_{2}{\bar{H}}_{2}$. The effects of the nine distinct composite diplotypes can be modeled through the traditional quantitative genetics model. Specifically, we use the Cockerham's orthogonal partition method [24] in which the genetic mean of an interaction model between blocks *s *and *t *can be expressed as

(1) μst=μ+asxs+atxt+dszs+dtzt+ iaaxsxt+iadxszt+idazsxt+iddzszt

where

$$\begin{array}{cccc} x_{s}= & \{\begin{array}{ll} \;\;1 & \text{for }H_{1}H_{1}\\ \;\;0 & \text{for }H_{1}{\bar{H}}_{1}\\ -1 & \text{for }{\bar{H}}_{1}{\bar{H}}_{1} \end{array} & z_{s}= & \{\begin{array}{ll} -1/2 & \text{for }H_{1}H_{1}\\ \;\;1/2 & \text{for }H_{1}{\bar{H}}_{1}\\ -1/2 & \text{for }{\bar{H}}_{1}{\bar{H}}_{1} \end{array} \end{array}$$

*x_t _*and *z_t _*can be defined similarly. With the above definition, *a*_*s*(*t*)_and *d*_*s*(*t*) _can be interpreted as the additive and dominance effects for the risk haplotype at block *s*(*t*); *i*_*aa*_, *i*_*ad*_, *i*_*da*_, *i*_*dd *_can be interpreted as the additive×additive, additive×dominance, dominance×additive, and dominance×dominance interaction effects between the two blocks, respectively.

Let *y*_*i *_denote a measured disease trait for subject *i*, which is dichotomous taking value 1 or 0, corresponding to affected or unaffected individual, respectively. Let *X_g _*denote a matrix of numerical codes corresponding to the two composite diplotypes as well as their interactions, and let *X_e _*denote a matrix of measured covariates, including the intercept as the first column. Let *x_ig _*and *x_ie _*denote the *i*^th ^row of *X_g _*and *X_e_*. Assuming that these factors influence the mean of a trait, so that their effects can be summarized by a function of linear predictors *η *= *X_g_β *+ *X_e_γ*, where *β *= [*a_s_*, *a_t_*, *d_s_*, *d_t_*, *i_aa_*, *i_ad_*, *i_da_*, *i_dd_*]*^T ^*contain regression parameters for the genetic effects of composite diplotypes on a disease trait; γ contain the effects of overall mean and the covariates. To simplify the notations, we also use *β *=[*β*_1_, *β*_2_, ...,*β*_8_]*^T ^*for the genetic effects in the equations below. Given a binary disease response, we can apply a conditional logistic model with the form

(2) $$\log\frac{p(y_{i}=1|x_{ig},x_{ie})}{p(y_{i}=0|x_{ig},x_{ie})}=x_{ig}\beta +x_{ie}\gamma$$

Compared to most non-parametric methods in detecting gene-gene interactions, such as the multifactor dimensionality reduction (MDR) method which only provides an interaction test [19], the above interaction model allows one to identify which ones are the risk haplotypes in two haplotype blocks, and to further quantify the specific structure and effect size of epistatic interactions between the two haplotype blocks. We argue that this model-based epistatic test provides biologically more meaningful results than a non-parametric method such as MDR.

### Likelihood function

We first introduce notations. Let *g_is _*and *g_it _*denote the observed genotypes in haolotype block *s *and *t *respectively for subject *i*. With the same numerical notation defined previously, we have *g_is_*, *g_it _*∈ {11/11, 11/12, 11/22, 12/11, 12/12, 12/22, 22/11, 22/12, 22/22}. Let *G_is _*and *G_it _*be the underlying composite diplotypes for *g_is _*and *g_it_*, respectively. We have$G_{is}\in \{H_{1}H_{1},H_{1}{\bar{H}}_{1},{\bar{H}}_{1}{\bar{H}}_{1}\}$ and $G_{it}\in \{H_{2}H_{2},H_{2}{\bar{H}}_{2},{\bar{H}}_{2}{\bar{H}}_{2}\}$. We further define *M*_1_, *M*_2_, *M*_3 _and *M*_4 _as four distinct genotype groups corresponding to the classification of phase (un)ambiguous haplotype blocks:

$$\begin{array}{l} M_{1}=\{i|g_{is}\neq 12/12\&g_{it}\neq 12/12\},\\ M_{2}=\{i|g_{is}=12/12\&g_{it}\neq 12/12\},\\ M_{3}=\{i|g_{is}\neq 12/12\&g_{it}=12/12\},\\ M_{4}=\{i|g_{is}=12/12\&g_{it}=12/12\}. \end{array}$$

To construct likelihood function, all three groups, *M*_2_, *M*_3_, *M*_4_, except group *M*_1_, involve phase ambiguity genotypes, hence need to be modeled with mixture distributions.

Define

$$\begin{array}{lllll} c_{si}= & \{\begin{array}{cc} 1 & G_{is}=H_{1}\bar{H}\\ 0 & G_{is}={\bar{H}}_{1}{\bar{H}}_{1} \end{array} & \text{and} & c_{ti}= & \pi \{\begin{array}{ccc} \begin{array}{c} 1\\ 0 \end{array} & \begin{array}{c} G_{it}\text{= }H_{2}{\bar{H}}_{2}\\ G_{it}\text{=}{\bar{H}}_{2}{\bar{H}}_{2} \end{array} & \text{for}\;i\;\in \;M_{2} \end{array} \end{array},M_{3,}M_{4}$$

We further define a set of the logistic regression functions for each genotype group as

$$\begin{array}{c} \pi_{M_{1}i}=p(y_{i}=1|x_{ig},x_{ie},i\in M_{1})=\frac{\exp(x_{ig}\beta +x_{ie}\gamma )}{1+\exp(x_{ig}\beta +x_{ie}\gamma )}\\ \pi_{M_{2}1i}=p(y_{i}=1|x_{ig},x_{ie},i\in M_{2},c_{si}=1),\\ \pi_{M_{2}0i}=p(y_{i}=1|x_{ig},x_{ie},i\in M_{2},c_{si}=0),\\ \pi_{M_{3}1i}=p(y_{i}=1|x_{ig},x_{ie},i\in M_{3},c_{ti}=1),\\ \pi_{M_{3}0i}=p(y_{i}=1|x_{ig},x_{ie},i\in M_{3},c_{ti}=0),\\ \pi_{M_{4}1i}=p(y_{i}=1|x_{ig},x_{ie},i\in M_{4},c_{si}=1,c_{ti}=1),\\ \pi_{M_{3}2i}=p(y_{i}=1|x_{ig},x_{ie},i\in M_{4},c_{si}=1,c_{ti}=0),\\ \pi_{M_{4}3i}=p(y_{i}=1|x_{ig},x_{ie},i\in M_{4},c_{si}=0,c_{ti}=1),\\ \pi_{M_{4}4i}=p(y_{i}=1|x_{ig},x_{ie},i\in M_{4},c_{si}=0,c_{ti}=0). \end{array}$$

Assuming independence between individuals, we construct the joint likelihood function as follows:

L=∑i∈M1log[πM1iyi(1−πM1i)1−yi]+∑i∈M2{csilog[πM21iyi(1−πM21i)1−yi]+(1−csi)log[πM20iyi(1−πM20i)1−yi]}+∑i∈M3{ctilog[πM31iyi(1−πM31i)1−yi]+(1−cti)log[πM30iyi(1−πM30i)1−yi]}+∑i∈M4{csictilog[πM41iyi(1−πM41i)1−yi]+csi(1−cti)log[πM42iyi(1−πM42i)1−yi]+(1−csi)ctilog[πM43iyi(1−πM43i)1−yi]+(1−csi)(1−cti)log[πM44iyi(1−πM44i)1−yi]}.

Because the phase ambiguous state *c_si _*and *c_ti _*are not observable, we treat them as missing data and use EM algorithm to estimate them iteratively (See below).

Variable selection methods such as LASSO [25] or adaptive LASSO [23] have been commonly applied when the number of predictors is large. These methods can achieve parameter estimation and variable selection simultaneously and have gained large popularity in genetic and genomic data analysis. Considering the large number of genetic parameters to be estimated in the model, we apply the adaptive LASSO to our model for its oracle property; namely, it performs variable selection and parameter estimation as if the true underlying model is known in advance [23]. Instead of maximizing the above log likelihood, we estimate the parameters by maximizing the log likelihood with the adaptive LASSO penalty.

(3) $$L'=-2L+\lambda \sum_{i}w_{i}|\beta_{i}|$$

where λ is a tuning parameter for the likelihood and penalty term, and is chosen by the minimum Bayesian Information Criterion (BIC); *ω *= (*w*_1_, *w*_2_, ..., *w*_8_) is a weight vector for the genetic effects *β*. When *w_j _*= 1 for every *j*, this leads to a general LASSO penalty. Although the general LASSO estimator may not be consistent, some data dependent weight vector *ω *is able to warrant the oracle property for the corresponding adaptive LASSO estimator. Specifically, one choice of *ω *is ω = 1/*β_OLS_*, where *β_OLS _*is the ordinary least square (OLS) estimator. This makes the adaptive LASSO estimate much more attractive than the general LASSO estimate [23].

### Missing data and the EM algorithm

The phase ambiguous genotypes lead to missing data. The currently developed algorithms LASSO or adaptive LASSO estimation can not be directly applied to maximize the penalized likelihood (3). However, this could be solved by applying an EM algorithm detailed as follows:

1) Initialize β, γ, and calculate $\pi_{i}=p(y_{i}=1|x_{ig},x_{ie})=\frac{\exp(x_{ig}\beta +x_{ie}\gamma )}{1+\exp(x_{ig}\beta +x_{ie}\gamma )}$ for subject *i*;

2) **E-step**: Estimate *c_si_*, *c_ti _*for subjects with phase ambiguous genotypes with *E*(*c_ji_*)by

$$E(c_{ji})=\frac{\phi_{j}\pi_{M_{k}1i}^{y_{i}}{\left(1-\pi_{M_{k}1i}\right)}^{1-y_{i}}}{\phi_{j}\pi_{M_{k}1i}^{y_{i}}{\left(1-\pi_{M_{k}1i}\right)}^{1-y_{i}}+(1-\phi_{j})\pi_{M_{k}0i}^{y_{i}}{\left(1-\pi_{M_{k}0i}\right)}^{1-y_{i}}},$$

for *i *∈ *M*_*k *_(*k*, *j*) ∈ {(2, *s*}, (3,*t*)}.

For i ∈ *M*_4_, we have

$$E(c_{si})=\frac{\phi_{s}\phi_{t}\pi_{M_{4}1i}^{y_{i}}{\left(1-\pi_{M_{4}1i}\right)}^{1-y_{i}}+\phi_{s}(1-\phi_{t})\pi_{M_{4}2i}^{y_{i}}{\left(1-\pi_{M_{4}2i}\right)}^{1-y_{i}}}{\Pi },$$

$$E(c_{ti})=\frac{\phi_{s}\phi_{t}\pi_{M_{4}1i}^{y_{i}}{\left(1-\pi_{M_{4}1i}\right)}^{1-y_{i}}+(1-\phi_{s})\phi_{t}\pi_{M_{4}3i}^{y_{i}}{\left(1-\pi_{M_{4}3i}\right)}^{1-y_{i}}}{\Pi },$$

where Π=ϕsϕtϕM41iyi(1−ϕM41i)1−yi+ϕs(1−ϕt)ϕM42iyi(1−ϕM42i)1−yi+(1−ϕs)ϕtϕM43iyi(1−ϕM43i)1−yi+(1−ϕs)(1−ϕt)ϕM44iyi(1−ϕM44i)1−yi

3) **M-step**: Update β,γ by maximizing the penalized log likelihood function (3);

4) Repeat step 1)-3) until convergence.

#### Computational algorithm for maximizing the penalized log likelihood

In the M step, parameters β, γ are updated by calculating LASSO estimate. The LASSO regression with continuous response has been well studied. Some very efficient algorithms have been proposed, such as the shooting algorithm and the LARS [26,27]. The estimation has been a challenge for the generalized linear model due to the non-linearity of the likelihood function, especially with an adaptive penalty term. No exact solution exists for parameter estimation in this setting. Here we propose a computational algorithm using a Gauss-Seidel method [28] to solve an unconstrained optimization problem. More detail about this method can be found in Shevade *et al*. [29]. To simplify the notations, we explain our method without environmental covariates.

We first derive the first order optimality conditions for the penalized likelihood (3). It is noticed that the penalized likelihood *L*' is piecewise differentiable. Following the notation in Shevade [29], denote *F_j _*= ∂(2 *L*)/∂*β_j_*. The first order optimality conditions ∂*L*'/∂*β_j _*= 0 could be achieved as follows:

$$\begin{array}{l} {{}_{}^{}}_{}^{}{{}_{}^{}}_{}^{}{{}_{}^{}}_{}^{}{{}_{}^{}}_{}^{}F_{j}{=}_{}^{}{}_{}^{}0_{}^{}{{}_{}^{}}_{}^{}{{}_{}^{}}_{}^{}{{}_{}^{}}_{}^{}{{}_{}^{}}_{}^{}if_{}^{}j=0\\ {{}_{}^{}}_{}^{}{{}_{}^{}}_{}^{}{{}_{}^{}}_{}^{}{{}_{}^{}}_{}^{}F_{j}=w_{j}\lambda_{}^{}{{}_{}^{}}_{}^{}{{}_{}^{}}_{}^{}{{}_{}^{}}_{}^{}if_{}^{}\beta_{j}>0,j>0\\ {{}_{}^{}}_{}^{}{{}_{}^{}}_{}^{}{{}_{}^{}}_{}^{}{}_{}^{}F_{j}=-w_{j}\lambda_{}^{}{{}_{}^{}}_{}^{}{{}_{}^{}}_{}^{}{}_{}^{}if_{}^{}\beta_{j}<0,j>0\\ {{}_{}^{}}_{}^{}-w_{j}\lambda \leq F_{j}\leq w_{j}\lambda_{}^{}{}_{}^{}if_{}^{}\beta_{j}=0,j>0 \end{array}$$

For the phase known genotypes, *F_j _*will have an explicit form as:

$$F_{j}=\sum_{i\in M_{1}}[y_{i}x_{ij}-\frac{\exp(\sum_{k}x_{ik}\beta_{k})}{1+\exp(\sum_{k}x_{ik}\beta_{k})}x_{ij}]$$

With the phase ambiguous genotypes, *F_j _*can be calculated accordingly with the mixture proportion *E*(*c_si_*)and *E*(*C_ti_*)that are estimated from E-step.

Based on the above conditions, we define

Violj=|Fj|if j=0=|wjλ−Fj|if βj>0,j>0=|wjλ+Fj|if βj<0,j>0=max(Fj−wjλ,−Fj−wjλ,0)if βj=0,j>0

Therefore, the optimal conditions could be achieved when *Viol_j _*= 0 for ∀*j*. For a given λ and *w_j_*, *j *= 1.....*p*, we further define *I_z _*= {*j*: *β_j _*= 0, *j *> 0}; and *I_nz _*= {0}∪{*j*: *β_j _*≠ 0, *j *> 0}. The detailed estimation procedure is given as:

1) Initialize *β_j _*= 0, *j *= 0, 1...... *p*;

2) While any *Viol_j _*> 0 in *I_z_*,

Find the maximum violator *V_k_*,

Update *β_k _*by optimizing *L*';

While any *Viol_j _*> 0 in *I_nz_*,

Find the maximum violator *V_l_*,

Update *β_l _*by optimizing *L*',

Until no violator exists in *I_nz_*;

Until no violator exists in *I_z_*

For computational precision purpose, the condition *Viol_j _*> 0 is relaxed to *Viol_j _*> 10^-5 ^in our computation.

This method is based on the convexity of the likelihood function. The computation procedure updates one *β_j _*at a time until all the optimality conditions are achieved. The algorithm is relatively efficient because it does not involve matrix inverse. The convexity condition warrants one and only one solution for each update (See additional file 1). Similar algorithm has been used in linear regression setting, commonly referred to as 'the shooting algorithm' [26], and in logistic regression setting for general LASSO [29]. The asymptotic convergence of this method for non-linear optimization problem has been proven in [[28], Ch.3Prop 4.1].

#### Risk haplotype selection

We treat each possible haplotype as a potential "risk" haplotype. The one with minimum BIC information defined below is chosen as the "risk" haplotype.

BIC=−2L+dlog(n)

where *d *is the number of non-zero parameters in the model and *n *is the total sample size.

### Results

#### Simulation study

We conducted a series of simulation with various scenarios to evaluate the statistical property of the proposed method. Within each block, the minor allele frequencies of the two SNPs were assumed to be 0.3 and 0.4 with a linkage disequilibrium *D *= 0.02. The simulation was conducted under different sample sizes (i.e., *n *= 200, 500, 1000)

Data were simulated by assuming one haplotype was distinct from the other ones for each block. Haplotypes were simulated assuming Hardy-Weinberg equilibrium. A disease status was simulated from a Bernoulli distribution with given genetic effects under different scenarios (Table 2). The intercept was adjusted to make the sample size ratio between cases and controls at approximately 1. Scenario S0 assumed no genetic effect at all. Other scenarios assumed different structure of genetic effects. Scenario S1 was an extreme case where all parameters were significant. The purpose of this simulation was to compare the selection power of different genetic parameters. Scenario S2 assumed that only one haplotype block has effects; Scenario S3 assumed both blocks had a genetic contribution to the disease phenotype without interaction between them; and Scenario S4 assumed both main and interaction effects between the two blocks. Data simulated with these configurations were subject to analysis with the proposed method. Results from 200 Monte Carlo repetitions were recorded.

**Table 2:** List of parameter values under different simulation designs

Scenario	***a***_**s**_	***a***_**t**_	***d***_**s**_	***d***_**t**_	***i***_***aa***_	***i***_***ad***_	***i***_***da***_	*i*_***dd***_
S0	0	0	0	0	0	0	0	0
S1	0.8	0.8	0.8	0.8	0.8	0.8	0.8	0.8
S2	0.8	0.8	0	0	0	0	0	0
S3	0.8	0.8	0.8	0.8	0	0	0	0
S4	0.8	0	0.8	0	0.8	0.8	0.8	0.8

**Figure 1:**
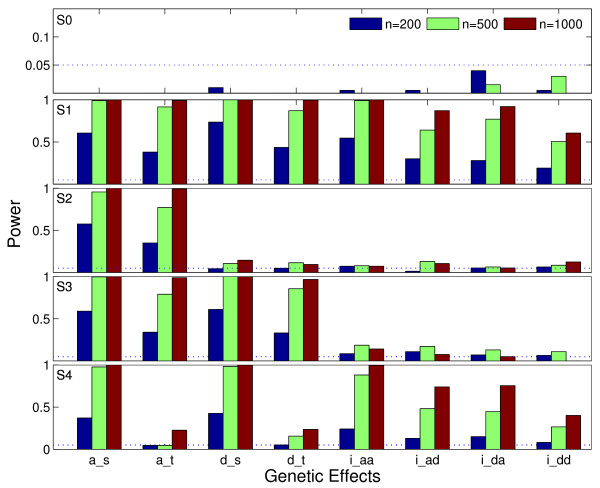
**The bar plot of variable selection results under different simulation scenarios**. Parameter values are listed in Table 2. The three sets of colored bars correspond to different sample sizes (Blue:200; Green:500; Red:1000). The horizontal dashed line indicates the nominal level of 0.05.

Figure 1 showed the results for variable selection under different simulation scenarios. For each genetic parameter, the three bars in color correspond to different sample sizes (see figure legend). The top figure corresponded to Scenario S0, in which the proportion of selection was equivalent to the false positive (or selection) rate. It can be seen that the false selection rates for all parameters were all under the nominal level of 0.05, indicating a good false positive control. For the other scenarios (S1-S4), the selection power increased as the sample size increased. Compared to S0, the selection rates for true negatives increased, but were also under reasonable control. Also as we expected, the selection power for the main effects was generally larger than the interaction effect (S1). Among the four interaction effects, the dominance×dominance effect performed the worst (S1 and S4). The simulation results also indicated that small sample size (*n *= 200) generally performed badly given the large number of genetic parameters to be estimated. Generally, at least 500 samples were required to achieve reasonable power to detect interactions.

#### A case study

We applied our model to a perinatal case-control study on small for gestational age (SGA) neonates as part of a large-scale candidate gene-based genetic association studies of pregnancy complication conducted in Chile. A total of 991 mother-offspring pairs (406 SGA cases and 585 controls) were genotyped for 1331 SNPs involving 200 genes. Maternal and fetal genome interaction was a primary genetic resource for SGA neonates. So we focused our analysis on identifying haplotype interactions between the maternal and fetal genome.

We first excluded SNPs that had a minor allele frequency of less than 5% or that did not satisfy Hardy-Weinberg equilibrium (HWE) in the combined mother and offspring control population by a Chi-squares test with a cut-off p-value of 0.001. We further used the computer software Haploview [30] to identify haplotype blocks for SNPs within each gene. Two tag SNPs were used to represent each block. A sliding window approach was applied to search for interactions between two blocks.

We picked two SNPs within each block and applied our model to study the main effects as well as the haplotype interaction effects between a mother and her offspring genome. By fitting our model as described in previous section and controlling other variables including maternal age and BMI, we successfully identified several SNP haplotypes with interaction effects through the adaptive LASSO logistic regression model. To ensure the significance, permutation tests of 1000 runs were further conducted to assess the significance. In each permutation test, the phenotypes were permuted and the model was fitted with different parameter estimate. An empirical p-value for effect *j *was calculated which is defined by

$$p-value\_j=\frac{\sum I_{|\beta_{perm,j}|>0}}{1000}$$

Results of the real data analysis were summarized in Table 3. Among the identified pairs, genes *HPGD *and *MMP9 *only showed main block effects. All the other five showed significant interaction effect. Permutation p-values confirmed the statistical significance of the detected effects. We used the maternal-fetal pairs to show the utility of our method. We could also do the analysis focusing on the fetal genome only. We thought an interaction between the maternal and fetal genome was more interesting, thus used this as an example.

**Table 3:** List of selected genes, corresponding "risk" haplotype structure, effect estimates and permutation p-values

SNP ID (allele)	Gene (region)	"Risk" haplotype	*a*_s_	*d*_s_	*a*_t_	*d*_t_	*i_aa_*	*i_ad_*	*i_da_*	*i_dd_*
9508994 (C/T)	PON1 (intron 1)	[TC]^M^	0	0	0	0	0	-0.45	0	0
20209376 (C/T)	PON1 (intron 5)	[CC]^O^						p* = 0.001		

659435566 (C/T)	NFKB1 (exon 12)	[CC]^M^	0	0	0	0	-0.33	0	0	0
659435702 (C/G)	NFKB1 (intron 22)	[TC]^O^					p* = 0.001			

22767327 (A/T)	FLT4 (intron 7)	[AT]^M^	0	0	0	0	0	-0.30	0	0
22175087 (C/T)	FLT4 (intron 8)	[TC]^O^						p* < 0.001		

1125300 (G/T)	SPARC (intron 3)	[TT]^M^	0	-0.38	0	0	0	0	0	0.245
1125290 (G/T)	SPARC (intron 5)	[TT]^O^		p* = 0.001						p* < 0.001

634841108 (A/C)	TIMP2 (intron 2)	[AG]^M^	0	0	0	0	0	0	0	0.68
634841123 (A/G)	TIMP2 (exon 3)	[CG]^O^								p* < 0.001

634018768 (A/G)	HPGD (promoter)	[AG]^M^	0	0	0.44	0	0	0	0	0
636105057 (A/G)	HPGD (promoter)	[GA]^O^			p* < 0.001					

17252653 (G/T)	MMP9 (intron)	[GC]^M^	0	0	0.53	0	0	0	0	0
17254821 (C/G)	MMP9 (exon 10)	[TC]^O^			p* < 0.001					

^M ^mother's "risk" haplotype information; ^O ^offspring's "risk" haplotype information

p* is the permutation p-value.

Our approach conducts the variable selection and effect estimation simultaneously, which allows us to have a direct biological interpretation for the mode of gene action. Here, we use gene *PON1 *as an example to illustrate the implementation of our model. In gene *PON1*, the selected risk haplotypes are [*TC*] for the mother and [*CC*] for the offspring. We find significant additive × dominant haplotype interaction effect. The two haplotypes separate all the mother-offspring pairs into three 'risk' groups with respect to the development of SGA:

R1={i|(GiM,GiO)=([TC]M[TC____]M,[CC]O[CC]O)|([TC]M[TC____]M,[CC]O[CC____]O)|([TC]M[TC____]M,[CC____]O[CC____]O)}R2={i|(GiM,GiO)=([TC]M[TC]M,[CC]O[CC]O)|([TC]M[TC]M,[CC____]O[CC____]O)|([TC____]M[TC____]M,[CC]O[CC____]O)}R3={i|(GiM,GiO)=([TC]M[TC]M,[CC]O[CC____]O)|([TC____]M[TC____]M,[CC]O[CC]O)|([TC____]M[TC____]M,[CC____]O[CC____]O)}

Following Eq. (1), we can see that R_1 _corresponds to the baseline reference group, R_2 _corresponds to the risk group with -1/2 interaction coefficient, and R_3 _corresponds to the risk group with 1/2 interaction coefficient. Correspondingly, the log odds of the disease development in each 'risk' group and the odds ratio (OR) between groups can be estimated by:

log(odds)=log(P(yi=1)P(yi=0))={μμ−iad/2 μ+iad/2i∈R1i∈R2i∈R3,OR={Referenceexp(−iad/2)=1.25exp(iad/2)=0.80 i∈R1i∈R2i∈R3

Other non-parametric methods, such as multifactor dimensionality reduction (MDR), have been shown to be successful for the identification of interaction effects in many studies. Because MDR can only be applied to studies with balanced case/control design, generalized MDR (GMDR) has been proposed as an extension to MDR [31]. GMDR maps phenotypic traits into residual scores through certain link functions under the generalized liner model setting, and further conducts SNP selection and testing based on the residual scores. To compare with our method, we applied GMDR to the data. The mother-offspring paired genotype data were used as input for GMDR, and a logistic link was used to calculate the residual scores.

In the example of *PON1*, SNP 20209376 (C/T) in the fetal genome was first selected by GMDR (p-value = 0.0107). SNPs were then paired with each other to identify potential significant pairwise interactions. Only SNP 9508994 (C/T) in the mother genome was found to interact with SNP 20209376 with marginal significance (p-value = 0.0547). More complex model were found to be non-significant (p-value = 0.1719 and p-value = 0.3770 for 3 SNP and 4 SNP model, respectively). Even though GMDR indicated a maternal-fetal interaction between these two SNPs, it did not provide an estimation of the genetic effect and the underlying interaction mechanism between the SNPs.

#### Model extension

Our method has been illustrated with two SNPs only. The model can be easily extended to more than two SNPs. When three or more SNPs are involved in each haplotype block, Cui *et al*. [12] gave an explicit derivation for possible "risk" haplotype structure. In fact no matter how may SNPs are involved, three possible composite diplotypes can be constructed as illustrated by Cui *et al*. [12]. The only challenge for this extension is to deal with the number of heterozygous loci. For example, when three SNPs are considered in a block, there are a total of seven possible phase-ambiguous genotypes. In a single block haplotype analysis, there could be four mixture distributions when constructing the likelihood function. When we consider interactions between two blocks, there are a total of 16 possible mixture distributions in the likelihood function. This will, however, definitely increase the programming challenge and the computing burden. Fortunately, the increaes of the mixture components will not affect the number of parameters to be estimated. We still have four main effects and four interactions, as these parameters are defined based on the "risk" haplotype structure.

Another possible solution to the challenges mentioned above is to do a sliding window search with each window covering two SNPs at a time. This is similar to the sliding window haplotype analysis commonly applied in some software such as PLINK.

### Discussion and Conclusions

Although it has been reported that gene-gene interaction plays a major role in genetic studies of complex diseases, the detection of gene-gene interaction has been traditionally pursued on a single SNP level, i.e., focusing on single SNP interaction. Intuitively, SNP-SNP interaction can not represent gene-gene interaction because single SNPs cannot capture the total variation of a gene. Thus, extending the idea of single SNP interaction to haplotype interaction could potentially gain much in terms of capturing variations in genes. The proposed method defines gene-gene interaction through haplotype block interactions and offers an alternative strategy in finding potential interactions between two genes. We argue that the definition of haplotype block interaction could provide additional biological insights into a disease etiology, compared to a single SNP-based interaction analysis.

One of the advantages of our method is in grouping, hence reducing data dimension. By mapping genotypes to composite diplotypes, the data dimension is significantly reduced. Then we can use Bayesian information criterion to select potential "risk" haplotypes [12]. The selection of "risk" haplotype renders another advantage of the method. We can identify significant haplotype structures and further quantify its main and interaction effects. This greatly enhances our model interpretability and biological relevance.

Our simulation study showed that our method has reasonable false positive control and selection power for the genetic parameters. As we expected, the interaction effects have lower selection power compared to the main effects. As sample size increases, we are able to achieve an optimal power for the interaction effects. Another novelty of the method is the modeling of the "risk" haplotype, which leads to the partition of composite diplotypes. No matter how many SNPs are involved, it always ends up with three types of composite diplotypes. Thus, the number of genetic parameters is always fixed regardless of the number of SNPs. The only cost is the search for possible "risk" haplotypes through a larger parameter space.

We applied our method to a SGA study data set. Several SNP pairs were selected with either main or interaction effects. The permutation test confirmed the statistical significance of the selected effect. Our findings confirmed other findings of gene selection in the literature. Gene *PON1 *was previously reported to be associated with preterm birth, which is one of the potential genetic resources leading to SGA [32]. Gene *FLT4 *had been found to be association with the growth of human fetal endothelia cells and early human development [33,34]. Gene *HPGD *was also reported being involved in human intrauterine growth restriction [35]. Gene *MMP9 *had been suggested to be related with placenta function [36]. These evidences strongly indicated the biological relevance of our method.

We also identified potential interaction effects for several additional genes, including *NFKB1*, *SPARC *and *TIMP2*. To our knowledge, no experimental evidence has been reported for these genes regarding the biological function related to fetal development or SGA. However, we found that each of these genes had been suggested to be involved in many biological pathways. Studies indicated that gene *NFKB1 *was functionally related to stress-impaired neurogenesis and depressive behavior [37], myelin formation [38], and adipose tissue growth [39]. Gene *SPARC *had been suggested to be associated with angiogenesis and tumor growth [40] and the progression of crescentic glomerulonephritis [41]. Gene *TIMP2 *was reported to be related to myogenesis [42] and the progression of cerebral aneurysms [43]. Further replicate studies are needed to confirm the biological relevance of these genes to SGA.

### Authors' contributions

ML performed the analysis and wrote the manuscript; RR collected the data; WF participated in the design and manuscript writing; YC conceived the idea, designed the model and wrote the manuscript. All authors read and approved the final manuscript.

## Supplementary Material

Additional file 1 **Strict convexity of the log likelihood function**. The file contains the proof of strict convexity of the log likelihood function. Click here for file
