# Supplementary material for: Mapping Haplotype-haplotype Interactions with Adaptive LASSO
Source: BMC Genet. 2010 Aug 27;11:79. doi: 10.1186/1471-2156-11-79 (PMC2946267; doi:10.1186/1471-2156-11-79)
Supplement: Additional file 1 — Strict convexity of the log likelihood function. The file contains the proof of strict convexity of the log likelihood function. [file 1471-2156-11-79-S1.DOCX]

**Strict Convexity of the log likelihood function**

First assuming there are no phase ambiguous genotypes, the log likelihood function can be expressed as:

$$\begin{matrix} L=\log\{\prod_{i=1}^{n} [\frac{\exp(\sum_{j=1}^{p} x_{ij}\beta_{j})}{1+\exp(\sum_{j=1}^{p} x_{ij}\beta_{j})}]^{y_{i}}[\frac{\exp(\sum_{j=1}^{p} x_{ij}\beta_{j})}{1+\exp(\sum_{j=1}^{p} x_{ij}\beta_{j})}]^{1-y_{i}}\} \\ =\sum_{i=1}^{n} y_{i}\sum_{j=1}^{p} x_{ij}\beta_{j}-\sum_{i=1}^{n} \log(1+\exp(\sum_{j=1}^{p} x_{ij}\beta_{j})) \end{matrix}$$

Therefore,

$$\begin{matrix} \frac{\partial L}{\partial\beta_{j}}=\log\{\prod_{i=1}^{n} [\frac{\exp(\sum_{j=1}^{p} x_{ij}\beta_{j})}{1+\exp(\sum_{j=1}^{p} x_{ij}\beta_{j})}]^{y_{i}}[\frac{\exp(\sum_{j=1}^{p} x_{ij}\beta_{j})}{1+\exp(\sum_{j=1}^{p} x_{ij}\beta_{j})}]^{1-y_{i}}\} \\ =\sum_{i=1}^{n} y_{i}x_{ij}-\sum_{i=1}^{n} [\frac{\exp(\sum_{j=1}^{p} x_{ij}\beta_{j})}{1+\exp(\sum_{j=1}^{p} x_{ij}\beta_{j})}x_{ij}] \end{matrix}$$

Furthermore,

$$\frac{\partial^{2}L}{\partial\beta_{j}^{2}}=-\sum_{i=1}^{n} [\frac{\exp(\sum_{j=1}^{p} x_{ij}\beta_{j})}{1+\exp(\sum_{j=1}^{p} x_{ij}\beta_{j})}x_{ij}]$$

For $\forall j,\exists i,1\leq i\leq n,s.t.x_{ij}>0$, therefore

$$\frac{\partial^{2}(-2L)}{\partial\beta_{j}^{2}}=2\sum_{i=1}^{n} [\frac{\exp(\sum_{j=1}^{p} x_{ij}\beta_{j})}{1+\exp(\sum_{j=1}^{p} x_{ij}\beta_{j})}x_{ij}]>0$$

It follows that $-2L$ is a strict convex function. With phase ambiguous genotypes, the convexity is not changed by linear operators. The strict convexity is a sufficient condition for minimizer uniqueness [44].
